# Control of Cucumber Powdery Mildew Using Resistant Cultivars and Organic Agricultural Materials

**DOI:** 10.4014/jmb.2409.09030

**Published:** 2024-11-28

**Authors:** Min-Jeong Kim, Yong-Ki Kim, So-Hyang Park, Jong-Ho Park, Sung-Jun Hong, Chang-Ki Shim

**Affiliations:** 1 Organic Agricultural Division, National Institute of Agricultural Sciences, RDA, Wanju 55365, Republic of Korea; 2 Technology Service Division, National Institute of Agricultural Sciences, RDA, Wanju 55365, Republic of Korea; 3 Department of Animal Husbandry and Sanitation, Jangsu 55634, Republic of Korea

**Keywords:** Bordeaux mixture, cucumber resistant cultivar, garlic extract, loess-sulfur mixture, powdery mildew

## Abstract

This study evaluated the efficacy of various inorganic materials, biological control agents, organic agricultural materials (OAMs), and essential oils for controlling cucumber powdery mildew (CPM) under greenhouse conditions. Treatments included silicate, bicarbonate, copper sulfate, sulfur-based compounds, *Bacillus subtilis* KB-401, *Bacillus amyloliquefaciens* M27, Bordeaux mixtures, chitosan combined with oxidated copper salt, chitooligosaccharides, and essential oils such as castor and rapeseed oil with lecithin. Results demonstrated that Bordeaux mixtures, particularly Bordeaux Mixture I, and sulfur-based compounds provided the most reliable CPM control among inorganic materials. Bacillus strains KB-401 and M27 achieved sustained disease suppression, suggesting their value in integrated pest management (IPM). Chitosan combined with oxidated copper salt showed high efficacy, making it a promising candidate for long-term control. Among OAMs, loess-sulfur mixtures proved especially effective in preventive applications, achieving comparable results to resistant cultivar 'Saronsamcheok'. Essential oils, including castor and rapeseed oil, also exhibited significant CPM suppression potential, although repeated applications may be necessary for sustained control. These findings highlight the potential of these environmentally friendly treatments as viable components of an IPM strategy for managing CPM in cucumber crops. Further studies are recommended to optimize combinations and application timing for enhanced efficacy.

## Introduction

Cucumber (*Cucumis sativus* L.) ranks among the most commonly cultivated crops in greenhouses globally. Fungal diseases present a significant challenge in commercial crop production, and powdery mildew is among the most damaging diseases affecting cucumbers. In South Korea, powdery mildew, primarily caused by *Sphaerotheca fusca* is a widespread issue that leads to severe economic losses and reduced yields [1-3].

Powdery mildew is less damaging than downy mildew, but when the disease develops sufficiently on the leaves to cover the surface of the leaves, cucumber growth is decreased, and photosynthesis is suppressed, thereby lowering the yield and quality (size, color, sugar content) of cucumber [4-6]. CPM is over-wintered in the diseased residue, and if the environment is suitable in the following spring, it produces numerous conidia spores and is spread by wind, insects, and splashing water. Temperature, relative humidity, and air circulation are well-known influential factors in the occurrence of powdery mildew. Unexpectedly, greenhouse cultivation is more favorable for disease occurrence in 20°C-30°C temperature and a relative humidity of 95% or more [7].

Nunez-Palenius *et al*. [8] point out that CPM occurs rapidly when the temperature is 24°C-30°C, relative humidity is 80%-95%, and there is no rainfall. Furthermore, the CPM pathogen (conidia) disperses well so that control by the cultural method is ineffective. Sivapalan [9] report that spore germination on the water’s surface is better than in the water, while the appressorium formation is better in the water’s than on the water surface. This demonstrates that humidity plays an important role in the pathogenesis of CPM. Douglas [10] mention that it is necessary to widen the planting distance in a cultural way, well ventilate in the facility, reduce the relative humidity to 93% or less, and implement drip irrigation to control CPM.

Powdery mildew spreads quickly under favorable conditions, with infection developing within 3–7 days as conidia are produced and disseminated [11]. Common management strategies include the use of disease-tolerant cultivars, fungicides, and biological agents [8]. Although fungicides are often highly effective, resistance to these treatments can emerge within powdery mildew populations, making alternative control methods increasingly important [12]. A variety of alternative products and integrated management approaches are therefore necessary to prevent resistance and ensure long-term control of the disease.

While evaluating the control efficacy of chemical pesticides against watermelon powdery mildew, Keinath and Dubose [13] suggest that there is a negative correlation between the AUDPC (Area under the disease progress curve) values and marketable yields, and that it is a proper epidemiological parameter by which to evaluate the treatment effects of chemical pesticides [14].

Environmentally friendly fungicides have been increasingly suggested as alternatives to synthetic fungicides, particularly in organic farming practices. Among these, several options for controlling cucumber powdery mildew include potassium bicarbonate, sulfur, neutralized phosphorous acid (NPA) [15-19], emulsified oil [20], and various biological control agents. NPA, known for eliciting plant defense mechanisms [21], has been reported as an effective control for powdery mildew caused by *Podosphaera xanthii* [19] and *Oidium* sp. [22] in greenhouse-grown cucumbers. Its use in managing powdery mildew and other crop diseases has gained popularity [18]. Cerkausskas and Ferguson [23] use the AUDPC as the epidemiological parameter while evaluating the effectiveness of new pesticides, biocides, and natural substances on CPM under greenhouse conditions. The disease incidence (diseased leaf area) and the disease severity (disease incidence) of CPM are mainly used as the epidemiological parameter by which to evaluate the resistance of the cucumber cultivars to CPM [17, 24-38].

Sulfur, one of the oldest known fungicides, has been used since the 19^th^ century to combat powdery mildew. It acts by disrupting the respiration of fungal spores and mycelia, making it effective both as a preventative and curative fungicide [39, 40]. Both NPA and sulfur are derived from naturally occurring plant nutrients, which suggests they pose minimal risk to human health and the environment [41, 42]. Despite their growing use, further research is needed to fully understand the agricultural and ecological impacts of these eco-friendly fungicides.

Silicon compounds have been recognized as biostimulants under recent EU regulations, while in other regions they are primarily used as soil amendments and occasionally classified as fertilizers. Since the 1990s, foliar applications of silicon compounds have become more common [43, 44].

Research on the use of sodium silicate for controlling powdery mildew shows promising results. Sodium silicate is commonly applied as a foliar spray to plants like cucumber, muskmelon, and zucchini squash. Studies indicate that sodium silicate forms a protective physical barrier on the leaf surfaces, which can effectively reduce powdery mildew infection by up to 87%. This reduction is attributed to both the barrier effect and the osmotic properties of the silicate, which inhibit fungal growth and conidia development. Additionally, silicon promotes plant resistance by enhancing the production of pathogenesis-related proteins, such as peroxidase and chitinase, although this effect is more pronounced with root applications than foliar sprays [43, 44]. In experiments with cucumber plants, sodium silicate significantly reduced colony formation of *S. fuliginea*, the fungus responsible for powdery mildew. The treatment was especially effective when applied at higher concentrations before fungal inoculation [45]. Silicon has been shown to improve disease resistance in plants, including cucumbers, by mitigating powdery mildew *S. fuliginea* [28]. Studies have reported a negative correlation between the silicon content in cucumber tissues and the severity of powdery mildew infections. Silicon works by strengthening plant tissues and acting as a physical barrier to fungal pathogens, which reduces the ability of the mildew to infect the plant. This has been confirmed in research by Menzies *et al*. [44], which demonstrated the effectiveness of silicon in decreasing the incidence of powdery mildew in cucumbers.

Sodium bicarbonate (NaHCO_3_), commonly known as baking soda, has been studied as an effective non-chemical method for controlling powdery mildew. Sodium bicarbonate increases the pH, creating an unfavorable environment for the growth of powdery mildew fungi, thereby reducing infection on plants. It is considered a safe and environmentally friendly option for organic farming. Research has demonstrated that sodium bicarbonate can be effective either on its own or when combined with adjuncts like vegetable oil or soap to reduce powdery mildew occurrence. However, excessive concentrations can cause phytotoxicity, so careful management of dosage and timing is essential for optimal results [46-49].

If resistant cultivars against CPM are not selected, it necessary to control them is using the cultural method and organic agricultural materials. Therefore, in order to effectively control CPM, a comprehensive approach, such as the use of cultural methods, the removal of infectious agents, adjustment of planting distance, and balanced fertilization and the application of organic agricultural materials (potassium bicarbonate, neem oil, garlic oil, milk, sulfur, and copper), including the cultivation of resistant cultivar, must be implemented [5, 26, 50-56].

Seo *et al*. (54) investigated the efficacy of garlic oil in controlling powdery mildew in greenhouse conditions. Their study demonstrated that garlic oil significantly reduced cucumber powdery mildew (*Sphaerotheca fusca*) and tomato powdery mildew (*Erysiphe cichoracearum*), achieving control rates of 70.0-74.6% and 71.2%, respectively. Singh *et al*. [57] explored the antifungal effects of ajoene, a garlic-derived compound, against *Erysiphe pisi*, the causal agent of pea powdery mildew.

In addition, many researchers have developed techniques using antagonistic microorganisms as a method to control CPM [33, 36, 58, 59]. DeBacco [10] reported that it is possible to reduce the incidence of powdery mildew by treating milk under open field condition. Hansen [60] mentions cultural control techniques for CPM, including the removal of inoculum sources, the prevention of overgrowth, and the proper fertilization of nitrogen. Several studies have shown that biological control methods are effective in managing cucumber powdery mildew [61-63]. The use of bio-agents has been successful in reducing the disease by inhibiting conidial germination, slowing mycelial growth, and decreasing the percentage of infected leaf area [2, 63, 64]. While, Soh *et al*. [37] reported that CPM can be reduced by mixing plant extracts (pine oil and tea saponin) with copper hydroxide, Wenneker and Kanne [65] reported that potassium bicarbonate successfully inhibits CPM. Yeo *et al*. [38] reported that plant extracts, such as extracts of *Rheum undulatum*; wettable sulfur powder; neem oil; and microbial formulations, such as *Ampelomyces quisqualis* 94031 and *B. subtilis* Y1336, are effective against *Cucurbitaceae* powdery mildew during the eco-friendly cultivation stage.

This study was conducted to select resistant cultivars and organic agricultural materials for controlling CPM in vivo. Moreover, the suppressive effect of resistant cultivars on CPM was evaluated by comparing the disease incidence of resistant cultivars with those of susceptible cultivars sprayed with a suspension of organic agricultural materials. Finally, we confirmed alternative effects of the selected resistant cucumber cultivars on organic agricultural materials.

## Materials and Methods

### Selection of Cucumber Powdery Mildew (CPM) Resistant Cultivars

In 2015, 88 commercial cucumber cultivars were used to select the cultivars resistant to CPM. The disease severity (diseased leaf area) of CPM was investigated in the greenhouse, plastic film house, and open field naturally infested with CPM, at the National Institute of Agricultural Sciences, Jeonju, Jeollabuk-do. Disease investigation was carried out when disease incidences reached a peak, which occurred on March 19^th^ in the greenhouse test, June 19^th^ (spring cultivation) and September 13^th^ (autumn cultivation) in the plastic film house test, and May 20^th^ (spring cultivation) in the open field test. The trial for resistant cultivar selection in the greenhouse test was a completely randomized design with three replications, which in the plastic film house and open field tests, the trials were randomized block designs with one replication and three replications, respectively.

### Suppressive Effects of Inorganic Materials on CPM

In order to evaluate the suppressive effect of resistant cultivar cultivation on CPM, 'Saeronsamcheok' as a resistant cultivar and 'Misobaekdadagi' as a susceptible cultivar were used in the plastic film house naturally infested with CPM to comparatively investigate the diseases development mode. A variety of inorganic materials were tested, including sodium silicate at different concentrations, sodium bicarbonate, montmorillonite, Bordeaux mixtures (copper sulfate + calcium oxide), lime sulfur (sulfur + calcium oxide), and sulfur in various formulations. The materials were applied to cucumber plants using the dilution ratios indicated in Table 1. Disease severity was evaluated by measuring the percentage of diseased leaf area at two time points, spring season (Diseased Leaf Area I) and autumn season (Diseased Leaf Area II), and the control efficacy of each treatment was calculated as Control Value I and II. OAMs were sprayed once, twice, and three times at seven day intervals from the beginning stage of CPM, and OAMs used were as follows: Bordeaux mixture (20% Sulfuric sulfate, and 40%slaked lime, diluted 100 times, Daeyu Co. Ltd.), sulfur-loess mixture (self-made goods: 25 kg sulfur powder, 15 kg Sodium hydroxide, 0.5 kg Loess, 1.5 kg natural salt, 0.5 kg Potash feldspar, 0.5 kg egg shell calcium powder, and 100 L Water, diluted 500 times, add 20 ml of brown rice vinegar based on 20 L of diluted sulfur mixture, and use after mixing evenly when spraying on cucumber), and garlic extract (self-made goods: 10% Garlic clove extract, 0.5% Canola oil, and 0.3% natural emulsifier).

In the case of loess-sulfur mixture, brown rice vinegar was added to the loess-sulfur mixture according to Shim *et al*. [52] method, and the pH was lowered to 10 or lower. The resistant cultivar Saeronsamcheok', was not treated with OAMs, but the susceptible cultivar 'Misobaekdadagi' were treated with OAMs. Disease incidences were investigated on Saeronsamcheok' and 'Misobaekdadagi' sprayed with the OAMs three days after the final OAMs treatment on the susceptible cultivar 'Misobaekdadagi'.

### Suppressive Effects of Antifungal Microorganisms and Their Eextracts on CPM

This study evaluated the efficacy of various antifungal microorganisms and microbial extracts in controlling cucumber powdery mildew (CPM) under greenhouse conditions. The suppressive effects of the treatments were assessed by measuring the diseased leaf area at two stages (Diseased Leaf Area I and II), and calculating the control efficacy at each stage (Control Value I and II). Several microbial treatments were tested, including *Bacillus amyloliquefaciens*, *B. subtilis* strains, *B. velezensis*, and others, in addition to microbial extracts. Each treatment was applied at specific dilution ratios as shown in Table 2, and the diseased leaf area was measured at two evaluation stages. Control efficacy was calculated by comparing the treated groups to the untreated control group.

### Suppressive Effects of Essential Oils on CPM

The efficacy of various essential oils and plant extracts for controlling cucumber powdery mildew (CPM) was evaluated under greenhouse conditions. The effectiveness of each treatment was measured by the reduction in diseased leaf area at two evaluation stages, and control values were calculated relative to the untreated control group. The essential oils and plant extracts tested included combinations of clove, castor, and orange oil, as well as individual treatments such as castor oil, *Cymbopogon martini* oil, and various other extracts. The oils were applied to cucumber plants at specified dilution ratios as detailed in Table 3. The disease severity was measured as the percentage of diseased leaf area in two evaluation stages (Diseased Leaf Area I and II), and the control efficacy was calculated as Control Value I and II.

### Suppressive Effects of Chitosan and Inorganic Materials on CPM

The efficacy of chitosan and its combinations with inorganic materials in controlling cucumber powdery mildew (CPM) was evaluated under greenhouse conditions. The treatments were assessed based on the diseased leaf area and control efficacy at two stages of disease development. Three treatments were tested: chitooligosaccharides, a combination of chitosan and oxidated copper salt, and a combination of chitosan and chitooligosaccharides. These treatments were applied to cucumber plants at the dilution ratios indicated in Table 4. Disease severity was measured as the percentage of diseased leaf area during two evaluation stages (Diseased Leaf Area I and II), and the control values were calculated relative to the untreated control group.

### Preventive and Curative Treatment Effect of the Selected OAMs on CPM

In order to investigate the preventive and curative effects of the egg yolk oil mixture and seven OAMs selected from the greenhouse tests on CPM, 'Eunsungbaekdadagi' plants were planted in a greenhouse where powdery mildew occurred the previous year, and OAMs were treated before and after the disease onset to investigate their control efficacy. The preventive effect of OAMs was evaluated as follows: When CPM occurred partially (1~2 lesions on 1~2 plants in the entire test field), OAMs were treated with the suggested concentration at seven-day intervals. Disease incidences (diseased leaf areas) were investigated after seven days of spray. Ten cucumber plants were planted in each treatment. The curative effect of OAMs was evaluated as follows: After three days of CPM occurrence, OAMs were treated with the suggested concentration three times at seven-day intervals. Disease incidences (diseased leaf areas) were investigated after seven days of spray. Ten cucumber plants were planted in each treatment.

### Development Mode of CPM on the Resistant Cultivar Compared with That on the Susceptible Cultivar Treated with OAMs

In order to compare the effects of resistant cultivar cultivation and OAMs treatment on the development of CPM, 'Saeronsamcheok' as a resistant cultivar and 'Misobaekdadagi' as a susceptible cultivar were used and OAMs, Bordeaux mixture, lime sulfur, and garlic extract were sprayed six times at seven-day intervals on the susceptible cultivar. A disease survey was conducted by investigating disease severity (diseased leaf areas) and the AUDPC were calculated by applying the disease incidence and investigation intervals of each lesion. This test was conducted in the plastic film house in which CPM naturally occurred.

### Investigation of Alternative Effect of Resistant Cultivar for OAMs

In order to determine the alternative effect of resistant cultivar on OAMs, three OAMs (Bordeaux mixture, loess-sulfur mixture, and sodium bicarbonate) were sprayed once, twice, and three times at seven-day intervals on susceptible cultivar ('Misobaekdadagi'), while no OAMs were sprayed on resistant cultivars ('Saeronsamcheok') in the plastic film house. A disease survey was conducted at the peak of the disease progress curve (June 10^th^), and the alternative effect of the resistant cultivar on OAMs was evaluated.

### Statistical Analysis

The SAS program (Statistical Analysis System, 7.13HF4, 2016) was used to screen the resistant cucumber cultivars and evaluate the effect of resistant cultivar cultivation and the application of OAMs on CPM. The *F*-value was calculated by one-way analysis of variance on the AUDPC, and the significance of treatments was evaluated by a t-test and Duncan's multiple range test (*P* = 0.05). The mean ± standard error of analysis of variance was determined and the statistical significance between treatments was tested using Duncan's multiple range test for the inhibitory effect of CPM on resistant cultivar cultivation and application of OAMs with different spraying times.

## Results

### Inhibitory Effect of Inorganic Materials on CPM

The results, summarized in Table 1, show that the various materials exhibited significant differences in their suppressive effects on cucumber powdery mildew. The effectiveness of sodium silicate varied with concentration. Sodium silicate V (90% concentration, 1:1000 dilution) showed the highest initial disease control, reducing the diseased leaf area by 82.9% in the first evaluation (Diseased Leaf Area I). However, its effectiveness declined in the second evaluation, where it achieved only 50% control (Control Value II). This material exhibited moderate efficacy, achieving 60.0% control in the first evaluation. Interestingly, its control efficacy improved significantly in the second evaluation, where it achieved an 84.0% reduction in diseased leaf area. While montmorillonite initially reduced the diseased leaf area by 24.6%, it exhibited a negative control value in the second evaluation, indicating that it was ineffective for sustained disease control. The Bordeaux mixtures demonstrated the highest levels of disease suppression across all treatments. Bordeaux Mixture I (20% copper sulfate + 40% calcium oxide) achieved near-complete disease control, with 97.1% control in the first evaluation and complete suppression (100%) in the second evaluation. Other Bordeaux mixtures (at different concentrations) also performed exceptionally well, maintaining control values above 85% throughout the study. Lime sulfur also provided strong disease suppression, with 92.7% control in the first evaluation and 84.0% in the second evaluation, making it one of the most effective sulfur-based treatments. Different sulfur formulations, particularly ultra-fine sulfur particles, were highly effective. Ultra-fine sulfur achieved an 85.1% control in the first evaluation and maintained high control efficacy (96.0%) in the second evaluation.

### Inhibitory Effect of Antifungal Microorganisms and Their Extracts on CPM

The results presented in Table 2 demonstrate significant differences in the suppressive effects of the antifungal microorganisms and microbial extracts. *B. subtilis* KB-401 showed the highest efficacy, achieving a remarkable 97.7% control in the first evaluation and maintaining a high control value of 95.5% in the second evaluation. The diseased leaf area was significantly reduced to 0.4% and 2.8% in the first and second evaluations, respectively, making this treatment the most effective among those tested. *Bacillus amyloliquefaciens* M27 strain exhibited strong disease suppression with an 84.6% control value in the first evaluation and a 92.0% control value in the second. The diseased leaf area was reduced to 2.7% in the first evaluation and 5.0% in the second evaluation, indicating sustained control over CPM development. Although *B. subtilis* DBB 1501 achieved high control (86.1%) in the first evaluation, its efficacy dropped significantly in the second evaluation, where the control value fell to 17.9%. The diseased leaf area increased from 2.4% to 51.3%, indicating a reduction in its long-term suppressive effect. *B. velezensis* NSB-1 showed moderate control efficacy, with 85.1% in the first evaluation but only 6.4% in the second, revealing a sharp decline in effectiveness over time. The diseased leaf area increased from 2.6% to 58.5%. Two microbial extracts were tested. Microbial Extract I showed strong initial control with an 88.6%reduction in diseased leaf area, though its control efficacy decreased to 49.3% by the second evaluation. Microbial Extract II performed less effectively, with control values of 74.3% and 38.8% in the first and second evaluations, respectively. *Streptomyces lavendulae*, this treatment reduced the diseased leaf area to 7.5% in the first evaluation, achieving a control value of 57.0%. Its efficacy improved in the second evaluation, reducing the diseased leaf area to 8.0% and increasing the control value to 87.2%, indicating delayed but effective control. *Paenibacillus polymyxa* AC-1 was among the least effective, with a diseased leaf area of 11.2% and a control value of 36.0% in the first evaluation. Although the control value improved slightly in the second evaluation (22.0%), its overall effectiveness remained limited. *B. licheniformis* NB109 + *Trichoderma atroviride* and *B. velezensis* G341 + *Lysinibacillus sphaericus* TC1, these combinations exhibited moderate control, with control values ranging from 63.3% to 81.3%in the first evaluation and 12.4% to 24.0% in the second evaluation, showing a decline in efficacy over time.

### Inhibitory Effect of Essential Oils on CPM

The results, as presented in Table 3, show a wide range of efficacy among the tested treatments.

Castor Oil exhibited the highest level of efficacy in the second evaluation, with a near-total reduction in the diseased leaf area (99.8% control). In the first evaluation, it reduced the diseased leaf area to 6.3%, with a control value of 64.0%, making it one of the most effective treatments overall. Rapeseed Oil + Lecithin, this combination was highly effective in both evaluations, with a control value of 93.7% in the first evaluation and 97.6% in the second. The diseased leaf area was reduced to 1.1% and 1.5% in the first and second evaluations, respectively, indicating sustained disease suppression. *Torilis Fructus* Oil was another effective treatment, achieving a 92.6%control value in the first evaluation. However, its efficacy declined in the second evaluation, where it reduced the diseased leaf area to 35.0%, corresponding to a 44.0% control value. *Cymbopogon martini* oil was also effective, with an 85.9% control value in the first evaluation and an 81.9% control value in the second. The diseased leaf area was reduced to 2.5% in the first evaluation and 11.3% in the second. The combination Soybean Oil and *Coptis chinensis* root extract showed good initial control with an 84.0% reduction in the diseased leaf area during the first evaluation. However, the control value dropped to 40.0% in the second evaluation, suggesting reduced long-term effectiveness. Rhubarb root extract exhibited moderate control, with a 42.9% reduction in diseased leaf area in the first evaluation and 89.3% control in the second. The diseased leaf area was reduced to 10.0% and 6.7%, respectively, indicating improved effectiveness over time. *Galla rhois* extract I showed moderate control in the first evaluation, reducing the diseased leaf area by 35.4%, but its effectiveness greatly improved in the second evaluation, achieving a 97.9% control value. In contrast, *Galla rhois* extract II had a lower initial control value (14.3%), but it performed well in the second evaluation, with a 93.9% control value. The combination *Thymus quiquecostatus* and *Sophora* extract achieved moderate efficacy, with a control value of 44.2% in the first evaluation and 9.8% in the second evaluation, indicating that its effectiveness decreased significantly over time. The extract combination *Rhus javanica* and *R. verniciflua* had limited efficacy, achieving a control value of only 8.3% in the first evaluation. In the second evaluation, it exhibited negative control (-36.0%), indicating an increase in disease severity relative to the control group. The combination *Mustard* and *Cinnamon* extract achieved moderate disease suppression, with a control value of 42.9% in the first evaluation and 52.0% in the second evaluation. The diseased leaf area was reduced to 10.0% and 30.0%, respectively.

### Inhibitory Effect of Chitosan and Inorganic Materials on CPM

The results in Table 4 show the suppressive effects of the tested treatments on CPM. The combination of Chitosan + Oxidated Copper Salt was the most effective treatment, achieving an 85.71% control value in the first evaluation and reducing the diseased leaf area to 2.5%. In the second evaluation, the control value remained high at 61.3%, with a diseased leaf area of 1.92%. This indicates that the chitosan and oxidated copper salt combination provided sustained disease suppression across both evaluation stages. Chitooligosaccharides treatment exhibited moderate effectiveness. It achieved a 45.71% control value in the first evaluation, reducing the diseased leaf area to 9.5%. In the second evaluation, it performed better, with a control value of 65%, indicating some improvement in disease suppression over time. The combination of Chitosan + Chitooligosaccharide was less effective than the other treatments, with a control value of 41.14% in the first evaluation. The diseased leaf area was reduced to 10.3%, but in the second evaluation, the control value dropped to 50%, and the diseased leaf area remained relatively high at 20.0%.

### Preventive and Curative Effects of Selected OAM on CPM

**Fig. 1:**
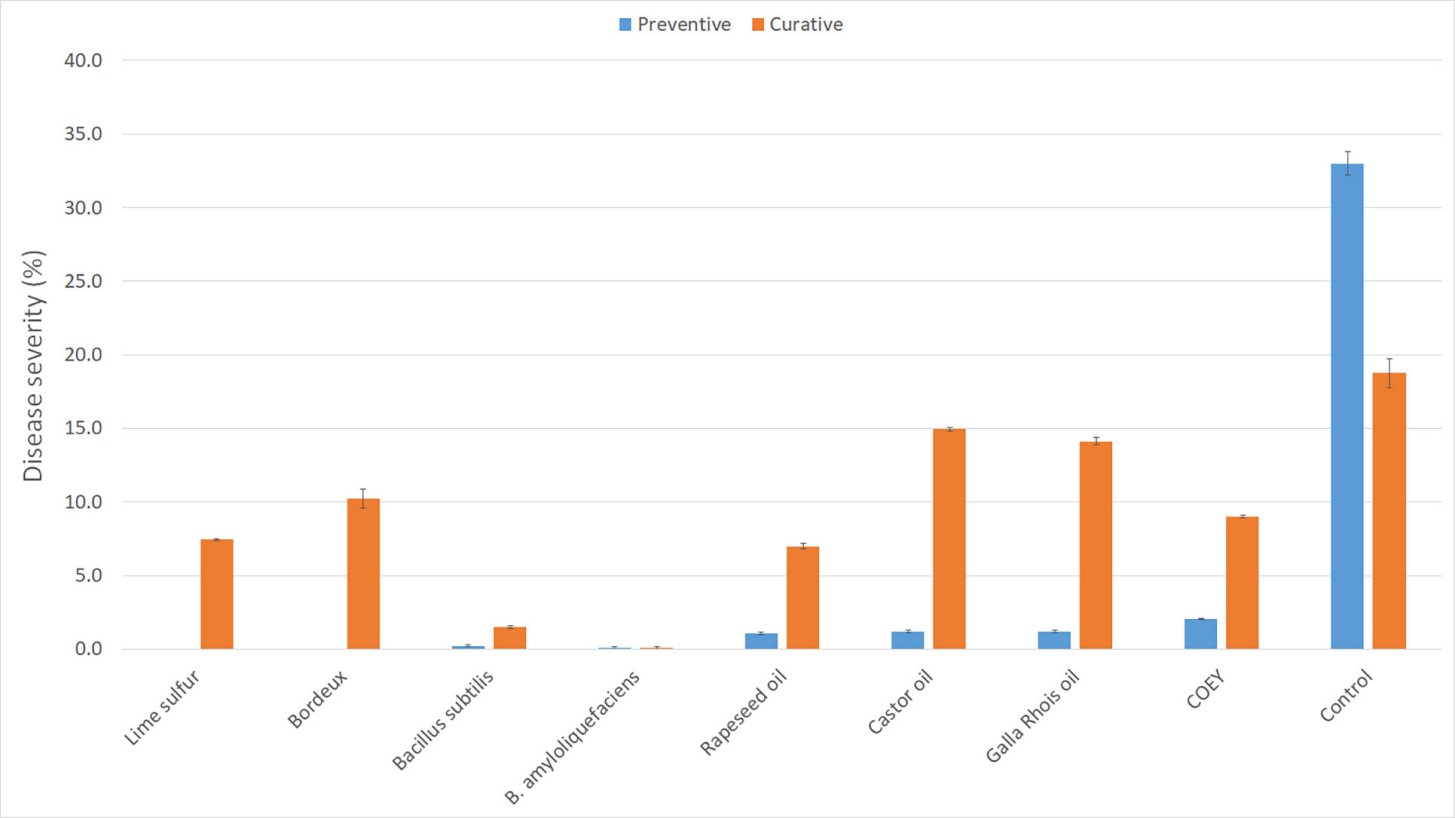
Preventive and curative effect of foliar-sprayed organic materials on the disease severity of cucumber powdery mildew (CPM) in the greenhouse. Cucumber used in this study was a susceptible cultivar ESBD for CPM. Error bars indicate standard deviation (*n* = 10) and different letter above the column indicates significant difference at 5% level by Duncan’s multiple range test.

When seven OAMs showing high control efficacy were preventively sprayed on the cucumber plants in the plastic film house, most of them showed high control efficacy against CPM. However, when they were curatively sprayed on the cucumber plants, except for two microbial formulations (*B. subtilis* KB-401 and *B. amyloliquefaciens* M27), most of them showed low control efficacy against CPM (Fig. 1).

### Suppressive Effect of Resistant Cultivar Cultivation and Application of OAMs on CPM

When OAMs were sprayed on cucumber plants, disease incidences were investigated and calculated, the AUDPC, in the resistant cultivar ('Saronsamcheok') cultivation plot was 38.4, and those in Bordeaux mixture, loess-sulfur mixture and garlic extract treatment plot were 46.1, 1.2 and 26.9, respectively. However. The AUDCP in the untreated susceptible cultivar plot was very high (1,056.8) compared with the OAMs application plot. Disease incidence was very low in the resistant cultivar cultivation plot and OAMs application plot and there was no statistical difference among the treatments (Table 5).

### Inhibition of CPM by Resistant Cultivar Cultivation and OAMs Sprayed with Different Application Times

**Fig. 2:**
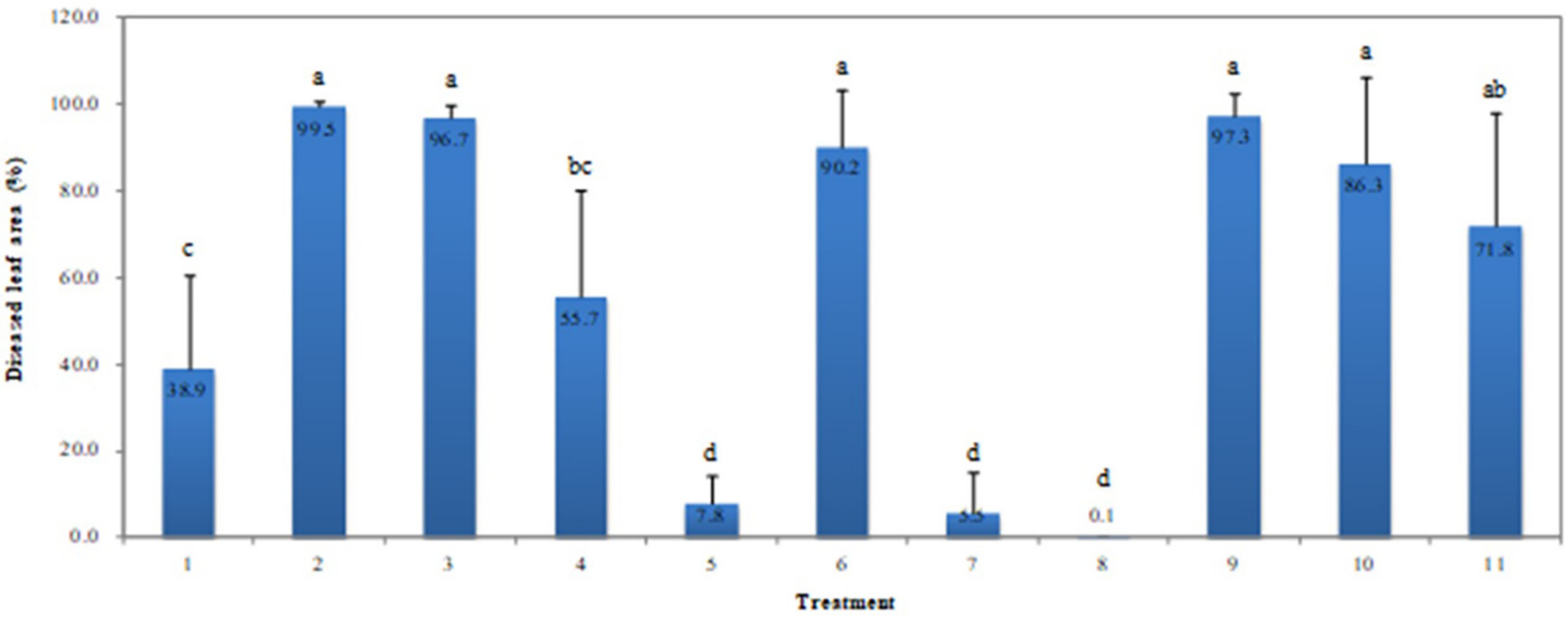
Disease severity of cucumber powdery mildew on resistant cultivar (CPM, bar 1) and susceptible cultivar (bar 2 to 11) for CPM sprayed at different application times with/without three organic materials in the plastic film house in Wanju County. Error bars indicate standard deviation (*n* = 30) and different letter above the column indicates significant difference at 5% level by Duncan’s multiple range test. Note: 1, Resistant cultivar ‘Saeronsamcheok’; 2, susceptible cultivar ’Misobaekdadagi (MBDD)’; 3, MBDD+ single application of Bordeaux mixture; 4, MBDD+two times application of Bordeaux mixture, 5, MBDD+three times application of Bordeaux mixture; 6, MBDD+single application of sulfur-loess mixture; 7, MBDD+two times application of sulfur-loess mixture, 8, MBDD+three times application of sulfur-loess mixture; 9, MBDD+single application of sodium bicarbonate; 10, MBDD+two times application of sodium bicarbonate; 11, MBDD+three times application of sodium bicarbonate.

OAMs were applied to evaluate the alternative effect of OAMs of resistant cultivar ('Saeronsamcheok') on the susceptible cultivar ('saeronsamcheok) in the plastic film house. As a result, when Bordeaux mixture, loess-sulfur mixture and sodium bicarbonate was applied to a susceptible cultivar, 1~2 times total and 1~3 times at seven-day intervals, it could not control CPM less than resistant cultivar cultivation. Whereas Bordeaux mixture and loess-sulfur mixture were applied three times total and 2~3 times at seven-day intervals to a susceptible cultivar, it could control CPM more than resistant cultivar cultivation. Disease incidence (diseased leaf area) on resistant cultivar, 'Saronsamcheok' was 38.9%, when Bordeaux mixture was applied once, twice, and three times at seven-day intervals on a susceptible cultivar, disease incidence measured 96.7%, 55.7%, and 7.8%, respectively. When loess-sulfur mixture was applied once, twice, and three times at seven-day intervals on a susceptible cultivar, disease incidence was 90.2%, 5.5%, and 0.1%, respectively. When sodium bicarbonate was applied once, twice, and three times at seven-day intervals on a susceptible cultivar, disease incidence was 97.3%, 86.3%, and 71.8%, respectively (Fig. 2).

## Discussion

The results indicate that Bordeaux mixtures and sulfur-based treatments are the most effective in suppressing cucumber powdery mildew under greenhouse conditions. Bordeaux Mixture I provided complete disease control in the second evaluation, suggesting that it is a highly potent fungicide for CPM management. Sulfur treatments, particularly ultra-fine sulfur, also demonstrated consistent and high efficacy. Sodium silicate and sodium bicarbonate showed moderate suppressive effects, though their long-term effectiveness may require repeated applications. Sodium silicate V exhibited strong initial disease control but declined in efficacy over time. Sodium bicarbonate, on the other hand, showed improved performance in the second evaluation, suggesting a possible delayed effect in disease suppression. Montmorillonite proved ineffective as a long-term control agent, with negative control values in the second evaluation. This suggests that while it may initially reduce disease incidence, it is not suitable for sustained powdery mildew management.

Tesfagiorgis [66] reported that water soluble silicone suppressed pumpkin powdery mildew by 32~70%. However, single treatment of five commercial products of sodium silicate showed very low control efficacy against CPM in this study. Further studies are needed to test the control efficacy of sodium silicates and the mixtures of microbial formulations showing high control efficacy in this study. Reuveni *et al*. [17] reported that micronutrient solutions including dipotassium hydrogen phosphate induces local and systemic protection against powdery mildew (*Sphaerotheca fuliginia*) in cucumber plants and can reduce disease incidence. We think the various OAM selected show high control efficacy in this study, and can be used to control CPM and establish a control strategy for the control of CPM. In the United States, control strategies using sulfurs, coppers, oils, and sodium bicarbonates showing high control efficacy have been set up at Purdue University and practically used to manage CPM in farmhouses [50].

The results indicate that *B. subtilis* KB-401 was the most effective microbial treatment, achieving the highest control values in both evaluations. This strain significantly reduced the diseased leaf area and maintained control over time, making it a promising candidate for integrated pest management (IPM) programs targeting CPM in cucumbers. *B. amyloliquefaciens* M27 also demonstrated strong and consistent suppression of CPM, with high control values in both evaluations. However, other *B. subtilis* strains, such as DBB 1501 and GB-0365, showed reduced efficacy in the second evaluation, suggesting that these strains may require repeated applications or a combination with other treatments for sustained control. The combination treatments, including *B. licheniformis* with *Trichoderma atroviride*, exhibited moderate efficacy but were less effective in the long term. These results suggest that further optimization of microbial combinations may be necessary to enhance their performance. The microbial extracts, particularly Microbial Extract I, showed strong initial control but diminished effectiveness over time, indicating that they may be useful in the early stages of disease development but may require supplemental treatments for long-term suppression.

Abdel-Kader *et al*. [24] reported that when *B. subtilis* and *T. harzianum* were treated alone and together with the disease resistance inducer (chitosan), they could effectively control CPM. Choi *et al*. [65] found that fourteen *Bacillus thuringiensis* isolates, known for their insecticidal and antifungal properties, effectively controlled several plant diseases. Notably, 12 isolates were effective against barley powdery mildew, and four also showed strong control against cucumber powdery mildew. Dik, *et al*. [57] reported that microbial formulations, *Ampelomyces quisqualis*, *Verticillium lechani* and *Sporothix flocculosa* were effective in controlling CPM and addition of silicone to the microbial formulations increased their control efficacy. Hijwegen [58] noted that for the control of CPM, *Tilletiopsis minor* was less effective under low humidity condition in the facility and relative humidity was very important for the expression of control efficacy of microbial fungicides. Rur *et al*. [2] reported that the combination of *Reynoutria sachaliensis* extract and *Yucca schidigera* extract as control agents for cucurbitaceous powdery mildew had excellent control efficacy and Konstantinidou-Doltsinis *et al*. [67] reported that a Milsana^R^ formulation made from *R. sachalinensis* extract inhibited tomato powdery mildew.

In contrast, the curative application of these OAMs, except for the two microbial formulations (*B. subtilis* KB-401 and *B. amyloliquefaciens* M27), showed significantly lower efficacy. This suggests that once CPM has established itself on cucumber plants, most of these OAMs are less effective in reversing the disease. The superior performance of the microbial formulations in curative scenarios indicates their potential as a more versatile option in managing CPM, likely due to their ability to actively target and suppress the pathogen even after infection has occurred. Nam *et al*. [36] reported that when *B. subtilis* KB-401 was preventively treated (before / in the early stage of disease development) to control CPM, it showed high control efficacy. Therefore, when OAMs were sprayed to control CPM, they should be preventively sprayed to obtain high control efficacy. Eight OAMs including two microbial formulations (*B. amyloliquefaciens* M27, *B. subtilis* KB-401), Bordeaux mixture, lime sulfur, rape seed oil, castor oil, *Galla rhois* oil, egg yolk and edible oil mixture showed high control efficacy, when they were sprayed preventively to control CPM. These OAMs also can be used to control CPM, if they are preventively sprayed on OAM in organic farmhouses. Kim *et al*. [30] reported that when ‘Chungkukjang’ containing various fermenting microorganisms like *B. subtilis* was sprayed on the cucumber plants, it could effectively control CPM. In this study, *B. subtilis* KB-401 showed high control efficacy against CPM, but the chitosan formulation showed comparatively low control efficacy.

The results demonstrate that castor oil and rapeseed oil + lecithin were the most effective treatments for controlling cucumber powdery mildew, with both achieving over 97% control in the second evaluation. Castor oil was particularly noteworthy for its near-complete disease suppression in the second evaluation. *Torilis fructus* oil and *Cymbopogon martini* oil also showed strong disease suppression, although their effectiveness declined in the second evaluation. These essential oils may require repeated applications or combination treatments to maintain their suppressive effects over time. Soybean oil + *Coptis chinensis* root extract and *Rhubarb* root extract demonstrated moderate efficacy, with rhubarb root extract showing improved control in the second evaluation. This suggests that these treatments may provide delayed but effective disease suppression. *Galla rhois* extract and *Thymus quiquecostatus* + *Sophora* extract exhibited varying degrees of efficacy, with *Galla rhois* extract I achieving high control in the second evaluation, while *Thymus quiquecostatus* + *Sophora* extract showed a significant decline in effectiveness over time. The combination of *Rhus javanica* + *R. verniciflua* extract was ineffective, and the negative control value observed in the second evaluation suggests that this treatment may exacerbate disease severity under certain conditions.

Singh *et al*. [56] showed that alone at a concentration of 25 mg/l completely inhibited conidial germination, while a 1,000 mg/l ajoene spray provided effective control of powdery mildew in a growth chamber, comparable to the commercial fungicides Sulfex and Bavistin. This suggests that ajoene has potential as an alternative treatment for managing powdery mildew in pea crops. Seo *et al*. [54] suggested that garlic oil has strong potential as a natural treatment for managing powdery mildew in crops under controlled environments. Bettiol [25] reported that milk showed control efficacy against pumpkin powdery mildew as high as that against chemical fungicides. Jannotti [51] reported that potassium bicarbonate, neem oil, sulfur formulations, and copper formulations effectively controlled powdery mildew. Keinath and DuBose [68] reported that the combined treatment of sulfur, fish oil and sesame oil effectively controlled powdery mildew in pumpkin seedlings. Jee *et al*. [26] reported that egg yolk and edible oil mixture could control cucumber downey mildew and CPM, and when mixed with egg yolk and an edible oil protected against cucumber and powdery mildew. Kim *et al*. [29] reported that diluted mayonnaises had control efficacy against CPM. Masheva *et al*. [34] reported that essential oils extracted from white mustard, hemp and wild yarrow were highly effective in controlling CPM and could be used in organic farmland. Sodium bicarbonate, which showed a relatively high inhibitory effect on CPM in the greenhouse test, showed very low control efficacy compared with loess-sulfur mixture and Bordeaux mixture. Although sodium bicarbonate is known to be widely used in organic disease management systems [5, 7, 50], its control efficacy was comparatively low in this study. We suspect that this due to the high disease pressure caused by frequent rainfall and the continuous cropping of cucumber. We concluded that of the three OAMs used, loess-sulfur mixture showed the highest control efficacy against CPM and can be used a control agent in farmer's field. This may be attributed to the fact that when the sulfur component was applied to the plant, it was evenly volatilized on the treated plant leaves so that it could act effectively across the target site.

The findings indicate that the combination of chitosan and oxidated copper salt was the most effective treatment for controlling cucumber powdery mildew under greenhouse conditions. This treatment consistently reduced the diseased leaf area and maintained high control values across both evaluation stages, suggesting its potential for long-term disease suppression. Chitooligosaccharides demonstrated moderate effectiveness, with an improvement in disease control during the second evaluation. This suggests that chitooligosaccharides may have a delayed effect in suppressing disease development, which could be useful for sustained management of CPM. The combination of chitosan and chitooligosaccharides was less effective overall, particularly in the second evaluation, where the control value dropped significantly. This indicates that while the combination may provide some initial suppression, it is not as effective as chitosan when combined with oxidated copper salt.

The results of this study demonstrate the significant suppressive effect of cultivating a resistant cucumber cultivar, combined with the application of organic and alternative materials (OAMs), on the incidence of cucumber powdery mildew (CPM). The resistant cultivar 'Saronsamcheok' showed an exceptionally low area under the disease progress curve (AUDPC) of 38.4, indicating its strong inherent resistance to CPM. This resistance was further enhanced by the application of OAMs, particularly the loess-sulfur mixture and garlic extract, which also exhibited low AUDPC values, reinforcing their effectiveness as protective treatments. As a result, the cultivation of resistant cultivar is considered to be an effective alternative to OAMs used for controlling CPM, and it could be effectively used together with OAMs in farmhouses. According to Yu *et al*. [69], some surfactants added for the control of plant diseases are known to have an additive effect on the dispersion of the active ingredients. Shnaider *et al*., [70] demonstrated the CRISPR/Cas9 technology to induce mutations in the CsaMLO8 gene in the susceptible cucumber cultivar 'Ilan,' creating two transgene-free mutant lines. These lines, which displayed high resistance to PM under semi-commercial conditions, offer a promising approach for quickly developing PM-resistant cucumber varieties across different genetic backgrounds. While several cucumber cultivars exhibit resistance to powdery mildew at higher temperatures (around 25°C), they tend to become susceptible when temperatures drop to around 20°C. Notably, the 'Kyuri Chukanbohon Nou 5 Go' cultivar has been reported to maintain high resistance at elevated temperatures, similar to the CS-PMR1 cultivar. Additionally, 'Kyuri Chukanbohon Nou 5 Go' shows slightly better resistance at lower temperatures compared to CS-PMR1, making it a valuable resource in breeding programs aiming for consistent resistance across varying environmental conditions [71, 72]. Further study for the selection of surfactants might be necessary to improve the control efficacy of garlic extract used in this study.

The study suggests that growers should consider both the selection of resistant cultivars and the strategic use of OAMs to manage CPM effectively. In cases where susceptible cultivars are used, increasing the frequency of OAM applications can significantly mitigate disease impact. Additionally, while sodium bicarbonate has some efficacy, it may need to be used in conjunction with other treatments or at higher frequencies to be as effective as other OAMs. Ary *et al*. [73] reported that *V. lechanii* 198499 showed antifungal activity against CPM. McGrath [6] reported that when sulfur formulations were foliar treated, the sulfur component was volatilized on the surface of the leaves and redistributed to the lower part of the leaves, and, as a result showed high disease control efficacy. Because the control efficacies of the treated OAMs were significantly different based on application times, their continuously application could be required to effectively control CPM during the disease development period. We think Bordeaux mixture and loess-sulfur mixture can be used to control CPM at organic farmhouses as well as conventional farmhouses. Overall, this research highlights the potential for integrating resistant cultivars with optimized OAM application schedules as a comprehensive approach to managing CPM, offering a viable alternative to chemical fungicides and contributing to more sustainable agricultural practices.

## Conclusion

The efficacy of several inorganic materials, including silicate, bicarbonate, copper sulfate, and sulfur-based compounds, was evaluated for their ability to suppress cucumber powdery mildew (CPM) under greenhouse conditions. The treatments were assessed based on their effects on diseased leaf area and control values at two stages of disease development. In conclusion, Bordeaux mixtures, especially Bordeaux Mixture I, and sulfur-based compounds such as ultra-fine sulfur, offer the most reliable and effective control of cucumber powdery mildew under greenhouse conditions. These findings suggest that these materials could be valuable tools in the integrated management of CPM in cucumber crops.

In this study, *B. subtilis* KB-401 and *Bacillus amyloliquefaciens* M27 were the most effective treatments for controlling cucumber powdery mildew in greenhouse conditions. These strains offer high and sustained control over the disease and could be valuable components of an IPM strategy. Further research is needed to optimize the use of microbial combinations and extracts to improve long-term disease suppression.

Chitosan combined with oxidated copper salt was the most effective treatment for controlling cucumber powdery mildew in greenhouse conditions. This treatment achieved high levels of disease suppression and maintained its efficacy over time, making it a promising candidate for integrated disease management. Chitooligosaccharides also showed potential, particularly for long-term disease control, while the combination of chitosan and chitooligosaccharides requires further optimization to enhance its effectiveness.

This study focused on environmentally friendly control of cucumber powdery mildew (CPM) using resistant cultivar 'Saronsamcheok', organic agricultural materials (OAMs) like loess-sulfur mixture, and biological control agents such as *Bacillus subtilis* KB-401 and *Bacillus amyloliquefaciens* M27. Preventive applications of OAMs generally showed high efficacy, particularly with loess-sulfur mixture. When applied 2-3 times, these treatments effectively suppressed CPM in susceptible cultivars, demonstrating comparable or superior control compared to the resistant cultivar. The findings support the use of these strategies for sustainable CPM management.

Castor oil and rapeseed oil + lecithin were the most effective essential oil treatments for controlling cucumber powdery mildew under greenhouse conditions. These treatments offer sustained disease suppression and could be valuable components of an integrated disease management program. Other essential oils, such as *Cymbopogon martini* oil and *Torilis fructus* oil, may also provide effective control but may require repeated applications for long-term efficacy. Further research is needed to optimize the application of these oils and to explore their potential for use in combination with other treatments for enhanced disease control.

## Figures and Tables

**Table 1:** Suppressive effect of inorganic materials-based silicate, bicarbomate, copper sulfate and sulfur on the development of cucumber powdery mildew in the green house.

Treatment	Active ingredient (%)	Dilution ratio (times)	Diseased leaf area I (%)	Control value I (%)	Diseased leaf area II (%)	Control value II (%)
Sodium silicate I	50	1,000	7.0 ± 0.4 f	60.0 ± 2.0 i	70.0 ± 3.5 b	-12.0 ± 5.7 k
Sodium silicate II	85	1,300	7.1 ± 0.3 e	59.3 ± 1.8 j	67.0 ± 2.5 c	-7.2 ± 4.0 j
Sodium silicate III	60	1,000	7.7 ± 0.5 c	56.0 ± 2.8 l	92.5 ± 1.8 a	-48.0 ± 2.8 k
Sodium silicate IV	15	1,000	7.5 ± 0.5 d	57.1 ± 2.8 k	70.0 ± 3.5 b	-12.0 ± 5.7 k
Sodium silicate V	90	1,000	3.0 ± 0.3	82.9 ± 1.6 g	50.0 ± 1.4 f	20.0 ± 2.3 i
Sodium bicarbonate	90	500	7.0 ± 0.4 f	60.0 ± 2.4 i	10.0 ± 2.1 h	84.0 ± 3.4 f
Montmorillonite	100	1,000	13.2 ± 0.1 b	24.6 ± 0.8 m	70.0 ± 1.4 b	-12.0 ± 5.7 k
Bordeaux mixture (copper sulfate + calcium oxide) I	20+40	100	0.5 ± 0.1 n	97.1 ± 0.4 a	0 ± 0 m	100 ± 0 a
Bordeaux mixture (copper sulfate + calcium oxide) II	14.5+14.9	500	2.5 ± 0.3 i	85.7 ± 1.6 e	4.0 ± 0.3 j	93.6 ± 0.5 d
Bordeaux mixture (copper sulfate + calcium oxide) III	30+30	200	0.8 ± 0.1 m	95.4 ± 0.8 b	3.3 ± 0.2 k	94.7 ± 0.4 c
Bordeaux mixture (copper sulfate + calcium oxide) IV	12+20	200	3.8 ± 0.2 g	78.3 ± 1.2 h	45.0 ± 0.7	28.0 ± 1.1 h
Lime Sulfur (sulfur + calcium oxide)	22+14	50	1.3 ± 0.1 l	92.7 ± 0.6 c	10.0 ± 1.4 h	84.0 ± 2.3 f
Sulfur I	1.7	1,000	2.0 ± 0.2 k	88.6 ± 1.2 d	4.3 ± 0.1 i	93.1 ± 0.2 a
Sulfur II	10	1,000	2.5 ± 0.1 j	85.7 ± 0.8	11.3 ± 0.2 g	81.9 ± 0.3 g
Ultra-fine particles of sulfur	50	200	2.6 ± 0.1 i	85.1 ± 0.8 f	2.5 ± 0.3 l	96.0 ± 0.5 b
Control	-	-	17.5 ± 1.4 a		62.5 ± 1.8 d	-

^z^Means followed by the same letter are not significantly different at 5% level by Duncan’s multiple range test.

**Table 2:** Suppressive effect of antifungal microorganism and it’s extract on the development of cucumber powdery mildew in the green house.

Treatment	Active ingredient (%)	Dilution ratio (times)	Diseased leaf area I (%)	Control value I (%)	Diseased leaf area II (%)	Control value II (%)
*Bacillus amyloliquefaciens* M27	40	500	2.7 ± 0.1 i	84.6 ± 0.6 d	5.0 ± 0.7 k	92.0 ± 1.1 b
*Bacillus subtilis* GB-0365	93.8	300	3.8 ± 0.2 g	78.1 ± 1.0 f	48.8 ± 0 f	21.9 ± 0 g
*Bacillus subtilis* DBB 1501	20	500	2.4 ± 0.5 j	86.1 ± 2.9 c	51.3 ± 0 e	17.9 ± 0 h
*Bacillus subtilis* KB-401	10	400	0.4 ± 0.1 l	97.7 ± 0.8 a	2.8 ± 0.1 l	95.5 ± 0.2 a
*Bacillus velezensis* NSB-1	98	500	2.6 ± 0.2 i	85.1 ± 1.2 d	58.5 ± 1.3 c	6.4 ± 2.0 j
*B. subtilis* + *B. amyloliquefaciens*	45+45	1,000	10.5 ± 1.1 c	40.0 ± 6.1 j	74.8 ± 2.9 a	-19.6 ± 4.6
*Bacillus licheniformis* NB109 + *Trichoderma atroviride*	100	1,000	3.3 ± 0.2 h	81.3 ± 1.0 e	54.8 ± 1.8 d	12.4 ± 2.9 i
*B. velezensis* G341+	15+25	1,000	6.4 ± 0.3 e	63.3 ± 2.0 h	47.5 ± 0 g	24.0 ± 0 f
*Lysinibacillus sphaericus* TC1						
*Paenibacillus polymyxa* AC-1	98.55	200	11.2 ± 1.3 b	36.0 ± 7.3 k	48.8 ± 1.1 f	22.0 ± 0.7 g
*Streptomyces lavendulae*	100	250	7.5 ± 0.2 d	57.0 ± 1.0 i	8.0 ± 1.2 j	87.2 ± 2.0 c
Microbial extract I	70	500	2.0 ± 0.7 k	88.6 ± 4.0 b	31.7 ± 1.3 i	49.3 ± 2.1d
Microbial extract II	70	500	4.5 ± 0.9 f	74.3 ± 4.9 g	38.3 ± 1.2 h	38.8 ± 2.0 e
Control	-	-	17.5 ± 1.1 a		62.5 ± 1.8 b	

^z^Means followed by the same letter are not significantly different at 5% level by Duncan’s multiple range test.

**Table 3:** Suppressive effect of essential oils on the controlof cucumber powdery mildew in the green house.

Treatments	Active ingredient (%)	Dilution ratio (times)	Diseased leaf area I (%)	Control value I (%)	Diseased leaf area II (%)	Control value II (%)
Clove + castor + orange oil	12+9+3	1,000	10.5 ± 1.1 e ^z^	40.0 ± 6.4 h	42.5 ± 2.1 d	32.0 ± 3.3 j
Castor oil	92	1,000	6.3 ± 0.2 h	64.0 ± 1.2 e	0.1 ± 0 m	99.8 ± 0 a
*Torilis fructus* oil	10	500	1.3 ± 0.1 k	92.6 ± 0.8 b	35.0 ± 2.1 f	44.0 ± 3.4 h
*Cymbopogon martini* oil	16.7	2,000	2.5 ± 0.2 j	85.9 ± 1.0 c	11.3 ± 0.7 h	81.9 ± 1.1 f
Rapeseed oil + lecithin	60+1.25	200	1.1 ± 0.2 l	93.7 ± 0.9 a	1.5 ± 0.2 k	97.6 ± 0.3 c
Soybean oil + Coptis chinensis root extract	93+5	400	2.8 ± 0.3 i	84.0 ± 1.6 d	37.5 ± 1.1	40.0 ± 1.7 i
Rhubarb root extract	10	1,000	10.0 ± 0.9 g	42.9 ± 5.3 f	6.7 ± 0.8 i	89.3 ± 1.4 e
*Thymus quiquecostatus* + Sophora extract	0.1+30	1,000	9.8 ± 0.7 f	44.2 ± 4.2 g	56.4 ± 2.4 c	9.8 ± 3.9 k
*Galla rhois* extract I	35	500	11.3 ± 0.9 d	35.4 ± 5.3 i	1.3 ± 0.2 l	97.9 ± 0.3 b
*Galla rhois* extract II	35	1,000	15.0 ± 0.9 c	14.3 ± 8.1 j	3.8 ± 0.5 j	93.9 ± 0.8 d
*Rhus javanica* + *R. verniciflua* extract	55+25	500	16.1 ± 1.0 b	8.3 ± 1.2 k	85.0 ± 2.8 a	-36 ± 4.5 l
Mustard + cinnamon plant extract	22+28	500	10.0 ± 1.6 e	42.9 ± 9.3 h	30.0 ± 4.9 g	52.0 ± 7.9 g
Control	0	0	17.5 ± 1.1 a	-	62.5 ± 1.8 b -

^z^Means followed by the same letter are not significantly different at 5% level by Duncan’s multiple range test.

**Table 4:** Suppressive effect of chitosan and inorganic materials on the control of cucumber powdery mildew in the green house.

Treatment	Active ingredient (%)	Dilution ratio (times)	Diseased leaf area I (%)	Control value I (%)	Diseased leaf area II (%)	Control value II (%)
Chitooligosaccharides	5	500	9.5 ± 0.14 c ^z^	45.71 ± 1.4 b	65 ± 1.41 a	4.0 ± 2.26 c
Chitosan+oxidated copper salt	17.5+6	500	2.5 ± 0.12 d	85.71 ± 1.2 a	61.3 ± 1.36 c	1.92 ± 2.18 b
Chitosan+Chitooligosaccharide	1.5+1.5	1,000	10.3 ± 0.75 b	41.14 ± 7.5 c	50 ± 1.58 d	20.0 ± 2.53 a
Control	-	-	17.5 ± 0.67 a		63.5 ± 1.49 b	-

^z^Means followed by the same letter are not significantly different at 5% level by Duncan’s multiple range test.

**Table 5:** Suppressive effect of resistant variety and organic materials (Bordeaux mixture and loess-sulfur mixture and garlic extract) against cucumber powdery mildew under plastic film house condition.

Treatment	Diseased leaf area (%) at different investigation date (Month/date)						AUDPC ^y^
	4/20	4/25	5/04	5/10	5/16	5/23	
Resistant cultivar-Saeronsamcheok	0.0 a ^z^	0.0 b	0.1 b	1.0 b	3.4 b	2.8 b	38.4 b^z^
Susceptible cultivar ‘MSBD ^v^’ / 4-6 type Bordeaux mixture	0.0 a	0.0 b	0.1 b	0.9 b	3.3 b	5.3 b	46.1 b
Susceptible cultivar MSBD/ loess-sulfur mixture^w^	0.0 a	0.0 b	0.0 b	0.0 b	0.1 c	0.1 c	1.2 c
Susceptible cultivar MSBD/ garlic extract^x^	0.0 a	0.1 b	0.1 b	0.1 b	1.1 c	5.2 b	26.9 b
Susceptible cultivar MSBD	0.2 a	0.6 a	10.2 a	26.8 a	73.7 a	95.6 a	1,056.8 a

^v^MSBD is a powdery mildew susceptible cultivar, 'Misobaekdadagi' .

^w^Loess-sulfur was made by as follows: 15 kg of sodium hydroxide and 25 kg of sulfur powder were put in 100 L of bowl and mixed evenly. And then 1.5 kg of natural sea salt, 500 g of clay powder and 500 g of potash feldspar were added in the mixture of sodium hydroxide and sulfur powder. First, the mixture was suspended in 30 L of water and reacted more than three times each other, and total volume of the mixture was fixed to 100 L. The formulated loess-sulfur mixture was diluted-sprayed five hundred times.

^x^Garlic extract was made as follows: Place the peeled 2 kg of garlic cloves and water in a blender beaker and grind them up using hand blender for 2~3 min, add 100 ml of canola oil and mix them again using hand blender for 2~3 min, add 60 ml of natural emulsifying agent and blend well using hand blender until they become creamy, pour the blended ingredients over the three layers of cheesecloth and then squeeze out them, dilute the prepared garlic clove extract and spray it evenly.

^y^AUDPC: Area Under Disease Progress Curve. zMeans followed by the same letter are not significantly different at 5% level by Duncan’s multiple range test.

^z^Means followed by the same letter are not significantly different at 5% level by Duncan’s multiple range test.
